# Pediatric endocarditis - a stone left after the pandemic cascade

**DOI:** 10.3389/fcimb.2024.1393315

**Published:** 2024-07-15

**Authors:** Ancuta Lupu, Alin Horatiu Nedelcu, Paula Diana Budescu, Elena Jechel, Iuliana Magdalena Starcea, Otilia Elena Frasinariu, Ileana Ioniuc, Minerva Codruta Badescu, Delia Lidia Salaru, Dragos Munteanu, Ruxandra Russu, Radu Andy Sascau, Cristian Statescu, Vasile Valeriu Lupu

**Affiliations:** Faculty of Medicine, “Grigore T. Popa” University of Medicine and Pharmacy, Iasi, Romania

**Keywords:** children, infective endocarditis, SARS-CoV-2 infection, immunosuppression, COVID-19

## Abstract

Infective endocarditis is a rare disease in children. The etiology is mainly bacterial. However, viral infective endocarditis, possibly related to severe acute respiratory syndrome coronavirus 2 (SARS-CoV-2), has also been reported. The pathophysiological principle of the connection between the two entities seems to be attributed to the transient immune deficiency of the body during the infection. Additionally, SARS-CoV-2 is reported in the literature as a direct cardiopathic virus. Therefore, the new coronavirus seems to have the ability to affect both the intact cardiac tissue and the previously damaged one both during the acute episode and at a distance from it. Consequently, we propose to review the main pathophysiological aspects of pediatric cardiac damage caused by SARS-CoV-2. The ultimate goal is to deepen existing knowledge, broaden the horizon of understanding and analysis regarding the systemic damage induced by viral infections, and strengthen an information base from which to start a meta-analysis. Next, we performed a non-systematized screening of the specialized literature with reference to cases of endocarditis in the pediatric population, reported in the period 2020–2023. From the total of articles found, we chose to include in the review a number of 6 case reports, including a number of 7 patients (5 children and 2 adolescents). Analysis of reports suggests that SARS-CoV-2 infection could play a role in the development of endocarditis, either directly through active infection or indirectly through a post-infectious immune response. Also, pre-existing conditions and complex medical history predispose to an increased risk of developing a severe, complicated form of endocarditis. Also, the lack of data on the vaccination history and the failure to categorize the infection depending on the type of antibodies (IgM or IgG) in some studies represent a major bias in the reports. The latter make it difficult to evaluate the influence of vaccination and the impact of acute versus chronic infection on the course of cases.

## 1 Introduction

Endocarditis remains a significant source of morbidity and mortality in children and adolescents (Gupta and Mendez, 2023). According to the latest estimates made by studying cohorts of pediatric patients in the USA and Australia, the incidence of infective endocarditis is 0.43 to 0.69 cases per 100,000 children, with an upward trend. About half of the included population has pre-existing heart damage. Among the most common causative pathogens, we note *Staphylococcus* aureus, followed by *Streptococcus*. Gupta S. et al. notes that, therefore, *Staphylococcus* seems to more frequently affect patients without pre-existing cardiac damage, in contrast to *Streptococcus*, the former predisposes to a longer duration of hospitalization and increased mortality (Gupta et al., 2017; Mahony et al., 2021). Infective endocarditis is a common complication that can occur in patients with congenital heart disease or rheumatic heart disease (Marelli et al., 2007; Warnes et al., 2008). Despite its high frequency, the current medical literature is poor in terms of reporting cases of viral endocarditis or precipitated by a previous viral infection (e.g., COVID-19) in the pediatric population. In this sense, two distinct cohorts of patients are defined, represented by children with endocarditis of unknown cause (possibly viral), respectively children with infectious bacterial/fungal endocarditis (possibly precipitated by a previous viral infection).

Immunocompromised individuals, those subjected to numerous invasive procedures, who have central catheters, and those institutionalized are the categories most exposed to the risk of developing this disease (Carceller et al., 2005). Only in small percentage of cases, infective endocarditis affects children who have no known chronic disease or congenital cardiac abnormalities (Vicent et al., 2022). The clinical signs of infective endocarditis in children are usually ambiguous and overlap with those of other more widespread diseases. Thus, identifying this condition is challenging and requires a significant degree of intuition on the part of the clinician to follow this diagnostic pathway (Tissières et al., 2003).

Continuing the previously stated directions, we note that post-COVID-19 endocarditis in the pediatric population is modestly cited in the literature. However, we aim to carry out this study to provide, in a structured manner, the latest information on the topic of interest. We identified the rarity of available data, their dispersion and the modest global experience of doctors with pathology as the “Achilles heel”, proceeding to perform the analysis to guide current and future diagnostic algorithms between multiple and non-specific manifestations. Also, we did not lose sight of the reporting rate of cases in the young adult population. By making an analogy between this, the reporting rate of post-COVID-19 endocarditis, but also of endocarditis independent of COVID-19 (the making/result of the specific test was not reported) in the pediatric population, we were able to conclude the main possible determining factors of the missed diagnosis.

## 2 State of the art

### 2.1 General guidelines regarding diagnosis

Currently, the modified Duke criteria are used to guide the diagnostic algorithm in infective endocarditis (Ferrieri et al., 2002). Few studies have evaluated the diagnostic performance of the criteria for pediatric infective endocarditis, with the Duke criteria being optimized mainly in adult patients (Martin et al., 1997; Ferrieri et al., 2002; Tissières et al., 2003). Although the modified Duke classification seems to be accurate in pediatric diagnosis as well, 12% of the possible cases of infective endocarditis (suggestive clinical picture plus echocardiographic changes) in them could not be classified as pathogenic based on blood cultures (Stockheim et al., 1998; Tissières et al., 2003). The diagnosis of infective endocarditis in pediatric patients can be more easily suspected if the child is in one of the risk groups and presents to the doctor with symptoms and signs closely related to this pathology. Imaging evaluation of the heart by transthoracic echocardiography, if necessary transesophageal, is a widely available exploration, and should be performed on all patients suspected of having infective endocarditis (Baddour et al., 2015; Habib et al., 2015). Vegetations, abscesses, and a new valvular prosthesis dehiscence are three significant features and are major criteria for the diagnosis of infective endocarditis. The minor diagnostic criteria refer to other signs and symptoms than those previously mentioned (**Table 1**).

**Table 1:** Diagnosis of Infective Endocarditis—The 2023 Duke-ISCVID Criteria for Infective Endocarditis adapted from (Fowler et al., 2023).

Diagnosis of Infective Endocarditis – Modified Duke Criteria	
Major criteria	Minor criteria
**I Microbiologic major criteria** **A. Positive blood cultures** 1. Blood culture positive for typical microorganism – isolated from two or more distinct sets of blood cultures (i.e., *Enterococcus, Streptococci viridans*, or *Staphylococcus aureus*) 2. Blood culture positive for microorganisms that occasionally or infrequently cause infective endocarditis obtained from three or more different blood culture sets **B. Positive results from laboratory testing** 1. Blood samples that have been positively tested by PCR or another nucleic acid-based method for *Bartonella* species, *Tropheryma whipplei*, or *Coxiella burnetii*. 2. A single blood culture or a *Coxiella burnetii* antiphase I IgG antibody titer > 1:800 3. IgM and IgG antibodies of *Bartonella henselae* and *Bartonella quintana* detected by indirect immunofluorescence assays (IFA) with the IgG titer greater than 1:800.	**I. Predisposition** **A. Previous history of infective endocarditis** 1. Prosthetic valve 2. Previous valve repair 3. Congenital heart disease 4. More than minor stenosis or regurgitation, no matter the cause **B. Endovascular cardiac implantable electronic devices** 1. Hypertrophic obstructive cardiomyopathy 2. Injection drug use
**II. Imaging major criteria** **A. Echocardiography and Cardiac Computed Tomography Imaging** 1. Echocardiography and/or Cardiac CT showing vegetation, leaflet/valvular aneurysm, leaflet/valvular perforation, abscess, intracardiac fistula, or pseudoaneurysm. 2. Compared to earlier imaging, a significant new valve regurgitation is observable on echocardiography. Pre-existing regurgitation is present but becomes worse or remains unchanging. 3. Compared to earlier imaging, the prosthetic valve has a new partial dehiscence. **B. (18F)FDG PET/CT Imaging** 1. Abnormal metabolic activity involving a natural or artificial valve, an ascending aortic graft (with accompanying valve involvement evidence), leads from an intracardiac device, or other prosthetic material.	**II. Fever** 1. Body temperature over 38°C
**III. Surgical major criteria** 1. During cardiac surgery, a direct observation providing evidence of infective endocarditis.	**III. Vascular phenomena** 1. Evidence, either clinically or radiologically, of artery emboli, cerebral or splenic abscess, septic pulmonary infarcts, intracranial hemorrhage, mycotic aneurysm, Janeway lesions, conjunctival hemorrhages, and purulent purpura.
	**IV. Immunologic Phenomena** 1. Rheumatoid factor 2. Roth’s spots 3. glomerulonephritis 4. Osler’s nodes
	**V. Microbiologic evidence, not meeting a major criterion** 1. Blood cultures that are positive for a bacterium compatible with infective endocarditis but do not fulfill the major criteria. 2. A single finding of a skin bacterium via PCR on a valve or wire without additional clinical or microbiological supporting evidence or a positive culture; PCR or other nucleic acid-based test for an organism consistent with infective endocarditis from a sterile body site other than cardiac tissue, embolus, or cardiac prosthesis.
	**VI. Imaging criteria** 1. Within three months after the implantation of intracardiac device leads, a prosthetic valve, prosthetic material, or ascending aortic graft abnormal metabolic activity measured by (18F)FDG PET/CT.
	**VII. Physical Examination Criteria** 1. If echocardiography is not available, auscultation may reveal new valvular regurgitation. It is insufficient if a pre-existing murmur worsens or changes.

Viral endocarditis is a rare condition in children (Del Pont et al., 1995). Several viral infections can potentially cause endocarditis in children, including enteroviruses, adenoviruses, coxsackieviruses, and cytomegalovirus (Douche-Aourik et al., 2005). These viruses can cause inflammation of the myocardium, which can, in turn, damage the heart valves and the development of endocarditis (Fong, 2009). Viral endocarditis is more prominent in animal models even though it is rarely encountered in humans (Burch et al., 1966; Burch and Tsui, 1971).

Symptoms of viral endocarditis in children can vary widely with the severity of the infection and generally include fever, fatigue, chest pain, difficulty breathing, rapid heartbeat, and swelling in the legs or abdomen (Mayo Clinic, 2023). Some children may have no symptoms. The diagnosis of viral endocarditis in children is challenging because the symptoms are often similar to those of other conditions, including bacterial endocarditis, rheumatic fever, Kawasaki disease, myocarditis, connective tissue disorders, and non-infective endocarditis.

### 2.2 COVID-19 and its cardiac involvement in children

Since the onset of the severe acute respiratory syndrome coronavirus 2 (SARS-CoV-2) pandemic, the majority of infected children were observed to exhibit no symptoms of the virus or only very mild illness (Nikolopoulou and Maltezou, 2022). COVID-19 is typically mild and self-limiting in children who were previously healthy, although cases of severe disease have been reported as well. One of the intensively studied pandemic paradoxes was the relationship between COVID-19 and systemic damage. Thus, the pandemic interfered with the evolution of autoimmune (e.g., systemic lupus erythematosus) or metabolic (e.g., diabetes) pathologies. At the same time, during this period there was an information peak regarding the micronutrition approach in pediatric respiratory pathology (e.g. bronchiolitis, pneumonia, COVID-19), with an emphasis on the immunomodulatory role of vitamin D. Heart involvement in multisystem inflammatory syndrome associated with COVID-19 in children (MIS-C) is a newly identified and challenging pathology that calls for prompt and accurate diagnosis, as well as the correct type of care (Kostik et al., 2022; Fotea et al., 2023; Lupu et al., 2023; Nicolae et al., 2023). Pediatric inflammatory multisystem syndrome—temporarily associated with SARS-CoV-2 (PIMS-TS) and presenting a phenotype mimicking Kawasaki disease (KD) (‘Kawa-COVID-19’)— was also identified in a small proportion of pediatric patients (Basu and Das, 2021; Mihai et al., 2023). Unlike Kawasaki disease, myocardial injury mainly defines MIS-C which facilitates the approach of differential diagnosis (Kostik et al., 2022). A trained innate immune system, a young and healthy immune system, and lower expression of the angiotensin-converting enzyme (ACE)-2 receptor (the target of SARS-CoV-2) gene may be reasons why children present milder COVID-19 symptoms than adults (Clark and Pathan, 2021). However, children can experience long-term COVID-19 side effects similar to those in adults, such as neurological and cardiac morbidity (Fotea et al., 2023). Severe acute infection with COVID-19 can have a negative impact on the cardiovascular, gastrointestinal, nervous, hematological, and renal systems (Axelerad et al., 2022; Kalyanaraman and Anderson, 2022; Mihai et al., 2022; Grigore et al., 2023). Children with COVID-19 rarely have major cardiac signs and symptoms, although cases of cardiogenic shock, myocarditis, pericarditis, and arrhythmias have been reported. Some children with severe heart disease from COVID-19 have died unexpectedly (Study: Cardiovascular Complications Following COVID-19 Are Uncommon in Children, Young Adults, 2022).

Damage to the cardiovascular system produced by this viral infection occurs through several mechanisms. The main mechanisms of cardiac tissue damage are represented by the direct effect of the virus, which has the ability to penetrate cells, hypoxia induced by lung injury, and the direct cardiotoxic effect of the drugs used in the therapy of COVID-19 (Fremed et al., 2020).

Viral RNA was found in 35% of myocardial infarctions in those infected with another strain of coronavirus (SARS-CoV). The direct viral aggression is based on the interaction of the virus with angiotensin-converting enzyme 2 (ACE2) receptors located on the surface of endothelial and endocardial cells (Song et al., 2012; Nishiga et al., 2020). Although it is known that SARS-CoV-2 enter the body primarily through an ACE2-mediated pathway (Zhang et al., 2020) there is no clear correlation between the degree of ACE2 expression in different organs, viral tissue tropism, and the high rate of infectivity. These findings imply SARS-CoV-2’s entry into cells may involve additional receptors or membrane proteins (Vukomanovic et al., 2021).

The pathophysiology of SARS-CoV-2 is characterized by an excessive generation of inflammatory cytokines (IL-6 and TNF-), which induces a systemic inflammatory response responsible for multiple organ and system failure. This mechanism has an immediate and major negative impact on the cardiovascular system. The most common cardiovascular complications reported in adult patients with COVID-19 include myocardial infarction, fulminant myocarditis rapidly progressing to decreased left ventricular systolic function, arrhythmias, venous thromboembolism, and cardiomyopathies mimicking ST-segment elevation acute myocardial infarction (STEMI) (Azevedo et al., 2021). Additionally, the renin–angiotensin–aldosterone system’s interactions with SARS-CoV-2 via the ACE2 receptor may exacerbate the inflammatory response and cardiac aggressiveness. This is specifically due to a minor molecular difference in the binding domain of the S-unit receptor in SARS-CoV-2 that increases its binding affinity to ACE2 (Chatterjee et al., 2023).

An indirect mechanism of myocardial damage is considered to be the increase in oxidative stress and, therefore, oxygen demand in the myocardial tissue following hypoxia induced by pre-existing lung damage (Chatterjee et al., 2023). There is also disruption of the immune system, marked by an increase in the neutrophil-lymphocyte ratio, in contrast to a decrease in suppressor T cells and T helper cells. The cytokine storm includes in its component the increase of granulocyte colony-stimulating factor, tumor necrosis factor (TNF)-α, interleukin (IL)-6, IL-2R, monocyte chemoattractant protein 1 together with interferon γ-inducible protein 10, chemokine (IL-8), macrophage inflammatory protein 1-α. To these is added the escalation of serum levels of C-reactive protein, procalcitonin, ferritin, erythrocyte sedimentation rate and fibrinogen. Cardiac markers (B-type natriuretic peptides, NT-proBNP and troponins) also register an important increase. Additionally, in most cases, an increase in the level of D-dimer can be noticed in contrast to the decrease in the albumin value (Chatterjee et al., 2023; Sirico et al., 2023). It is noted in the literature that the development of the type 2 inflammatory process associated with an increase in Th2 and eosinophils in the peripheral blood can be considered a favorable prognostic factor. They seem to play a key role in reducing the level of ACE2 expression on epithelial cells (Vasichkina et al., 2023). Major cardiac damage was attested by Octavius GS. et al. and Khairwa A. et al. in necroptic studies (Khairwa and Jat, 2022; Octavius et al., 2022).

**Figure 1:**
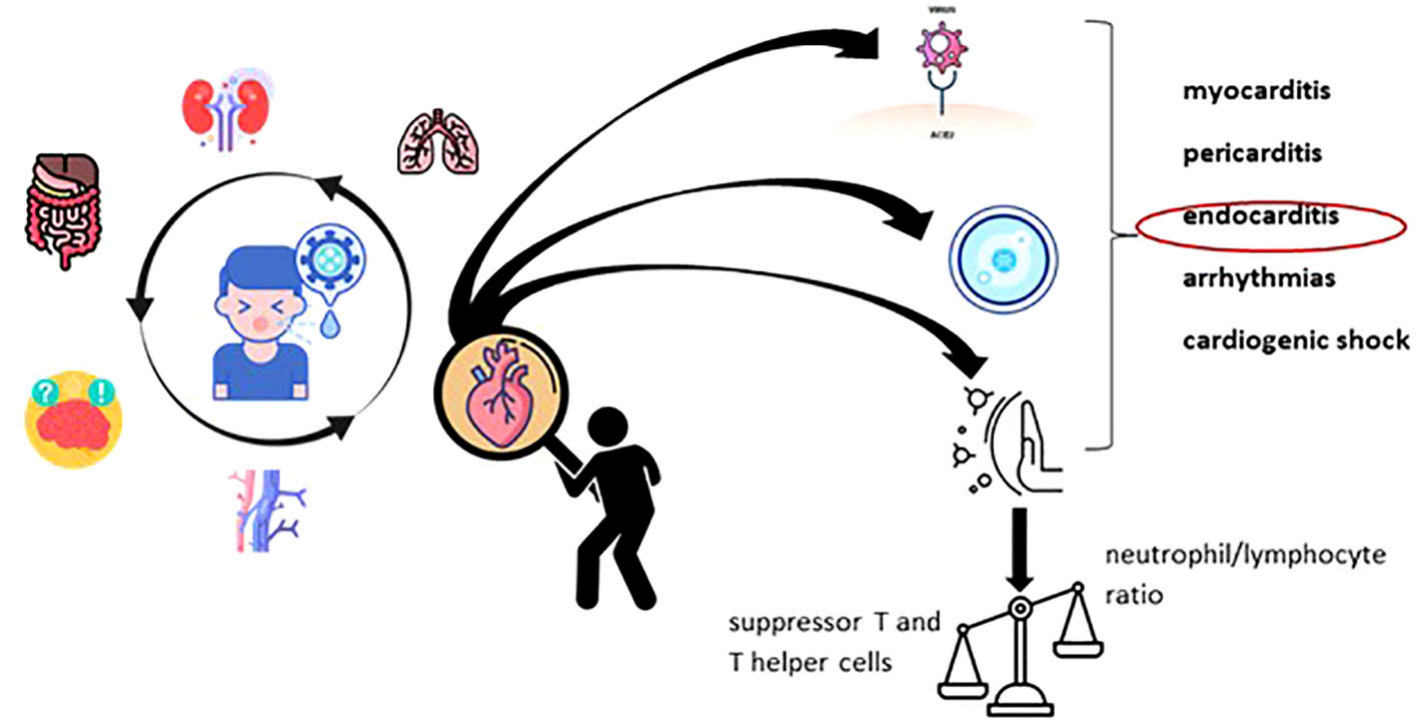
Systemic damage in the infection with COVID-19 - focus on the cardiac component.

By way of consequence, **Figure 1** presents the graphic illustration of the main disruptions induced by the Sars-Cov-2 virus, which dynamically determines cardiac damage. I want to arouse the interest of readers in terms of understanding, diagnosing and treating heart damage from viral diseases. Although the pandemic is over, humanity can always be put in a situation to counteract a virus with cardiac “tropism”.

## 3 Endocarditis in children with COVID-19 infection

### 3.1 Exposure of pediatric endocardial cases during the COVID-19 pandemic

There have been some reports on COVID-19-related cardiac complications in adults, including myocarditis and pericarditis. However, the incidence of these complications in in the pediatric population is much lower. We are on an upward slope of research regarding the relationship between COVID-19 and pediatric endocarditis. The crossroads between the two pathologies seems to be systemic inflammation. These findings feed the theories regarding the costimulation of the two. It is thus necessary to widen the horizon in terms of understanding the co-existence of the two. The essential consideration resides in the increased risk of developing COVID-19 (severe form) and endocarditis among children with pre-existing heart conditions (Rodriguez-Gonzalez et al., 2020). In this way, we want to take advantage of the upward trend in international reports on cases of pediatric heart disease during the pandemic period. We therefore challenge ourselves in collecting and centralizing reference data regarding the chosen research topic.

#### 3.1.1 Methods

We conducted an electronic search of international databases (e.g., PubMed, Embase, Google Scholar) from January 2020 to December 2023 (pandemic and post-pandemic reference period) and retrieved original studies investigating endocarditis in children infected with SARS-CoV-2. The search was carried out in an unsystematized manner. We used the following search terms: (“endocarditis”) AND [(“children”) OR (“pediatric”) OR (“paediatric”) OR (“child”)] AND [(“COVID-19”) OR (“coronavirus-19”) OR (“SARS-CoV-2”)]. To reduce the risk of data loss, which could induce bias, we did not limit our search to articles published or translated into English. The final goal is to create a physiopathogenic basis of reference in understanding the interaction. In the secondary plan, we want a summary analysis of the general conclusions from the literature necessary to guide a targeted direct, worthy of meta-analysis (e.g., the rate of missed diagnosis during the pandemic period, the incidence of endocarditis in the pediatric population vs. young adults vs. adults, the incidence of endocarditis in a structurally normal heart vs. a malformed heart or the incidence in vaccinated versus unvaccinated children).

#### 3.1.2 Results

The results of the non-systematized screening of the specialized literature were summarized in **Table 2**. They include six case reports, summing up a total of 7 patients, aged between 2 months and 18 years. We would like to specify that the inclusion in the report of an 18-year-old patient results from the combination of two considerations. Mainly, the authors consider the age of 18 as a boundary between the adult and pediatric period. Also, due to a low density of cases in the specialized literature, we consider the report valuable and necessary to be taken into account to avoid biases. Subsequently, both their conclusions and key points were developed in a narrative manner. The most frequent symptom was fever, found in 3 out of 4 patients. The paraclinical investigations highlighted both acute and previous infection with SARS-CoV-2. Furthermore, regardless of the result of the blood cultures -bacterial, fungal or unspecified pathogen-, the presence of endocarditis was confirmed in all cases by echocardiography and anatomopathological examination. Notably, only two patients had a medical history, while the other two patients developed infective endocarditis without having any other pathology apart from SARS-CoV-2 infection.

**Table 2:** Patients with endocarditis related to COVID-19.

Author	Age, gender	Acute/previous SARS-CoV-2 infection	Symptomatology	Type of endocarditis	Pathogen	Medical history
Nazrin T. et al., 2023 (Nazrin and Hassan, 2023)	2 year 3-month-old, male	Anti-COVID antibodies positive	Continuous, recurrent fever with prolonged duration; At present: fever for 7 days (highest peak of temperature was 104°F), associated with poor feeding and irritability	Bacterial endocarditis	vancomycin resistant *Staphylococcus aureus*	Child from consanguineous parents
	3 year 6-month-old, male	Positive SARS-CoV-2 antigen, identified by reverse transcriptase-polymerase chain reaction	High grade, irregular fever for last 4 months, erythematous rash over both upper and lower limbs	Bacterial endocarditis	Blood culture: *Staphylococcus Saphrophyticu*s Culture from the surgically removed sample: *Mycobacterium tuberculosis*	Congenital mild pulmonary valve stenosis Infective endocarditis (vegetation on pulmonary valve) which was treated with antibiotics and antifungal for four months (10.06.19 to 08.09.2019)
Ganguly M. et al., 2022 (Ganguly et al., 2022)	2-month-old, child	Anti-COVID antibody with high inflammatory markers	Hemodynamically significant ventricular tachycardia	Bacterial endocarditis	*Acinetobacter*	Unknown
Cinteza E. et al., 2022 (Cinteza et al., 2022)	7-year-old, male	IgG antibodies positive for SARS-CoV-2 (RT-PCR)	Watery stools and fever	Fungal endocarditis	*Cunninghamella* spp.	Beta-thalassemia
Khoshnood M. et al., 2021 (Khoshnood et al., 2021)	3-year- old, female	IgG antibodies for SARS-CoV-2 present	Recurrent fever of unknown origin	Bacterial endocarditis	Unknown	Down Syndrome, unbalanced atrioventricular canal, atrial septal defect, ventricular septal defect patch, hypoplastic right ventricle, pulmonary hypertension, and obstructive sleep apnea
Aikawa T. et al., 2021 (Aikawa et al., 2021)	18-years-old, male	Positive SARS-CoV-2 antigen, identified by reverse transcriptase-polymerase chain reaction	Acute dyspnea	Unspecified	Unspecified	Without medical history
Dolhnikoff M. et al., 2020 (Dolhnikoff et al., 2020)	11-year- old, female	Acute COVID-19 infection (SARS-CoV-2 RNA detected on a post-mortem nasopharyngeal swab)	Fever for 7 days, odynophagia, myalgia and abdominal pain	Unspecified	Unspecified	Without medical history

In 2023, Nazrin T. et al. reports two cases involving children aged 2 years and 3 months, respectively 3 years and 6 months.

The first case is a boy from consanguineous parents. He is hospitalized in the context of repeated febrile episodes, lasting 10–15 days. He currently has a high fever for about 7 days, loss of appetite and irritability. No history of respiratory symptoms or contact with a confirmed person with COVID-19. At presentation, the child is afebrile, moderately pale, tachypneic, tachycardic with a positive Kernig sign. Biologically, microcytic anemia, hypochromia, thrombocytopenia, increased inflammatory markers, increased D-dimer and ferritin and hypoalbuminemia were detected. Chest radiograph have objectified a right lower zone consolidation. Reverse transcription polymerase chain reaction (RT-PCR) for COVID-19 was negative, but COVID antibodies were positive (possible following a recent chronic infection, no vaccination data being reported). Echocardiography in 2D mode and color Doppler revealed vegetation at the level of the pulmonary, pulmonary insufficiency grade II, without stenosis and mild pulmonary arterial hypertension. Blood cultures revealed the presence of vancomycin resistant *Staphylococcus aureus*. Other cultures, bacteriological and imaging examinations were without pathological changes, thus excluding meningitis. Under treatment the symptoms gradually remitted. The dynamic ultrasounds have shown the reduction in the size of the vegetation. After 5 weeks of treatment, the authors reported the disappearance of vegetation (Nazrin and Hassan, 2023).

The second case is another boy, hospitalized for irregular, high-grade fever for the last 4 months, which was accompanied by an itchy erythematous rash both over the upper and lower limbs. The child was known to have mild congenital pulmonary valve stenosis and a history of infective endocarditis on the pulmonary valve. Presented recent history of contact with a positive COVID-19 patient. Biologically, microcytic hypochromic anemia, D-dimer and elevated ferritin are observed. He tested positive for COVID-19 by RT-PCR. 2D echocardiography and color Doppler revealed a large echogenic, irregular, diamond-shaped, homogeneous mass at the level of the pulmonary valve, mild pulmonary valve stenosis and a small aneurysmal dilatation, with normal cardiac function. Chest x-ray - right radiolucent area with the appearance of a calcified hilar lymph node. The patient underwent surgery to remove the vegetation, and biopsy revealed *Mycobacterium tuberculosis*. Thus, the treatment with antituberculosis drugs was initial (Nazrin and Hassan, 2023).

Previous, Ganguly M. et al. presented the case of a 2-month-old-child who required medical assistance due to hemodynamically significant ventricular tachycardia. Blood investigations revealed raised anti-COVID antibodies and high levels of inflammatory markers. However, it is not specified whether the COVID antibodies were of the IgM or IgG type. In addition, blood culture at admission was positive for *Acinetobacter*. The echocardiography illustrated a hyperechoic mass attached to the anterior mitral leaflet (Ganguly et al., 2022).

Another case of a 7-year-old male was identified by Cinteza E. et al. He was hospitalized for watery stools and fever. Forty-eight hours after the onset of symptoms, a nasopharyngeal sample was collected for SARS-CoV-2 (RT-PCR), and the results were negative, but IgG antibodies were positive. The patient presented coryza and rhinorrhea two weeks before hospital admission, symptoms that could be manifestations of acute COVID-19 infection. The electrocardiogram revealed pathological Q waves in DII, DIII, aVF, and V4–V6; a 2 mm depression in the ST segment in V5–V6; and negative T waves in DI and aVL. Furthermore, the echocardiography showed massive endocarditis that severely affected the tricuspid and mitral valves (multiple mitral and tricuspid valve vegetations were identified). Evidence of embolization in the kidney, spleen, lower limbs, brain, and lung was also present. Emergency heart surgery for mitral and tricuspid valves was performed. Polymerase chain reaction (PCR) performed on the vegetations confirmed *Cunninghamella* spp. as the etiology of the infection (Cinteza et al., 2022).

Similarly, Khoshnood M. et al. reported the case of a 3-year-old girl known to have Down Syndrome, an unbalanced atrioventricular canal, an atrial septal defect, a ventricular septal defect patch, a hypoplastic right ventricle, pulmonary hypertension, and obstructive sleep apnea who needed medical care for a recurrent fever of unknown origin. The SARS-CoV-2 nasopharyngeal PCR was negative, SARS-CoV-2 Ig G antibodies were present. The patient had a medical history of SARS-CoV-2 3 months before current admission to the hospital. The echocardiography showed a significant thrombus throughout the length of the atrial septal defect patch, extending across the plane of the atrioventricular valve and along the length of the ventricular septal defect patch with protrusions into the ventricle via the mitral valve. It was decided to attempt to remove the thrombus. The anatomopathological examination demonstrated the presence of granulation tissue formation, which was considered a marker of bacterial endocarditis (Khoshnood et al., 2021).

Aikawa T. et al. reports the case of an 18-year-old who returns for hospitalization complaining of acute dyspnea, unaccompanied by fever. The presentation occurs at an interval of 3 months after infection with the SARS-CoV-2 virus, confirmed by RT-PCR. RT-PCR for SARS-CoV-2 on admission was positive, while serological tests for other viral infections were negative. Electrocardiographically, a pattern of early repolarization in the precordial leads and ST-segment depression in lead III is objectified. Transthoracic echocardiography revealed only a subtle dyskinesia at the level of the anterior wall of the left ventricle. Biologically, cardiac troponin T showed high levels (642 ng/l), a fact that required an urgent computed tomography angiography. Post-contrast injection (10 minutes) a midwall delayed enhancement was identified in the left ventricular anterior wall. Serum C-reactive protein levels were negative throughout the evolution. The endomyocardial histopathology of the right ventricle revealed an aspect strongly suggestive of an endocarditis complicating myocardial fibrosis post COVID-19 (Aikawa et al., 2021).

The beginning of reports was marked by Dolhnikoff M. et al. They presented the case of an 11-year-old child who presented with fever, odynophagia, myalgia, and abdominal pain. MIS-C related to COVID-19 was diagnosed. She developed heart failure and died 1 day after admission to the hospital, SARS-CoV-2 RNA was detected in a post-mortem nasopharyngeal swab and in cardiac and pulmonary tissues by RT-PCR. The autopsy showed endocarditis, myocarditis, and pericarditis, with intense and diffuse tissue inflammation and necrosis of cardiomyocytes. Electron microscopy analysis of cardiac tissue revealed the presence of spherical viral particles in the extracellular compartment and within several cell types, including cardiomyocytes, capillary endothelial cells, endocardium endothelial cells, neutrophils macrophages, and fibroblasts. These particles matched the *Coronaviridae* family in terms of size and form (Dolhnikoff et al., 2020).

The previously described clinical cases illustrate various clinical presentations and dynamics of endocarditis among children and adolescents, assumed to be associated with SARS-CoV-2 infection. As can be seen, the onset of cardiac damage can be accompanied by persistent fever, skin rashes, difficulty breathing, loss of appetite, irritability, and rhythm disturbances. Most patients had a history of contact with people infected with SARS-CoV-2 or previously confirmed infection. Some showed symptoms suggestive of active or recent SARS-CoV-2 infection, while others tested positive for antibodies against the virus. Unfortunately, only in two situations was the reporting of anti-Covid 19 antibodies dependent on their type (IgM versus IgG). We can thus draw attention to a data reporting bias that prevents us from creating a distinctive profile of cases from the point of view of new infection or previous infection with COVID-19. Imaging and laboratory tests are the mainstay for diagnosing endocarditis and evaluating the extent of valvular lesions. In dynamics, different bacteria and even fungi have been identified as etiological agents, including *Staphylococcus aureus*, *Acinetobacter* or *Cunninghamella* spp. Also, part of the cases did not have the specified etiological diagnosis. The severity and course of endocarditis associated with COVID-19 can vary, from full recovery under appropriate treatment to serious complications. Thus, the importance of prompt diagnosis and management of endocarditis is reiterated, especially in a pandemic context. Further studies are needed to define the link between the two conditions, to understand and to provide the possibility of extrapolating the data in the future.

Comparing the cases in terms of immune status, vaccination history, congenital disorders predisposing to cardiological or immune phenomena, we observe the following:

- All cases have evidence of SARS-CoV-2 infection, either by the presence of antibodies or by positive RT-PCR tests. This suggests that SARS-CoV-2 infection could play a role in the development of endocarditis, either directly through active infection or indirectly through a post-infectious immune response.

- Congenital heart, genetic predispositions or immunological disorders (e.g., mild congenital stenosis of the pulmonary valve, beta-thalassemia, Down syndrome, septal defects, hypoplastic ventricle, pulmonary hypertension, obstructive sleep apnea) are present in a few cases and may predispose to endocarditis or other complications. Patients with preexisting conditions appear to be at greater risk of developing severe or complicated endocarditis.

- The lack of data on vaccination history makes it difficult to assess the influence of vaccination on these cases.

### 3.2 And yet, how high is the rate of missed diagnoses?

Currently, there is insufficient evidence to support the existence of a definite link between acute or previous infection with the SARS-CoV-2 and the onset of infective endocarditis in children. However, infection with SARS-CoV-2 can be considered a trigger for the development of cardiac complications because it produces a procoagulant status and promotes the development of a systemic inflammatory response in the body. Cardiac complications in children affected by COVID-19, such as myocardial and coronary involvement, are not uncommon and must be carefully recognized and continuously monitored (Sperotto et al., 2021).

During the SARS-CoV-2 pandemic, most children presenting to the hospital were suspected of being infected with COVID-19, which is why this was the first diagnosis that had to be confirmed or denied. For this reason, a number of illnesses could have been interpreted as infection with SARS-CoV-2 until PCR test result was received. In February 2022, Peña-Moreno A. et al. reported the case of a 10-year-old boy who was misdiagnosed with MIS-C. The boy presented a six-day history of fever, abdominal pain, vomiting, and fingertip pain and headache, symptoms that in the context of the SARS-CoV-2 pandemic were suggestive of COVID-19. Although the SARS-CoV-2 reverse transcription polymerase chain reaction and serology results were ultimately negative, MIS-C was initially suspected as the cause of the patient’s symptoms. Subsequent, echocardiography showed that the mitral valve had an echogenic lesion, and blood culture results turned positive for *Staphilococcus aureus* sensitive to methicillin (Peña-Moreno et al., 2022).

Early in the COVID-19 pandemic, when little was known about this new disease, the fear of contacting SAR-CoV-2 itself generated certain complications. Yeshayahu Y. reported a case of a 16-year-old patient with trisomy 21 but without known heart disease who at the beginning of the pandemic presented himself to the hospital with fever accompanied by knee and hip pain. Although a new heart murmur was detected during clinical examination of the patient’s heart, the family refused further investigations due to fear of contacting the SARS-CoV-2. Seven weeks after the initial visit, the child’s condition worsened. The patient then returned to the hospital where a wider panel of investigations was carried out. Ultimately, a diagnosis of endocarditis was made, and surgical intervention was found to be necessary. Notably, during the COVID-19 pandemic the referral of the pediatric population to the hospital suffered due to parents’ concern of exposing their children to the SARS-CoV-2 by visiting hospitals or leaving their homes, which led to the delay in the diagnosis of some major heart diseases (Yeshayahu, 2021).

Pediatric patients presenting with infective endocarditis either in acute SARS-CoV-2 infection or at a distance from the acute episode are a real challenge for physicians. There are few cases of infective endocarditis directly related to SARS-CoV-2 infection. However, numerous studies highlight that infection with this new virus produces a systemic inflammatory syndrome and a significant decrease in immunity (Pouletty et al., 2020; Kabeerdoss et al., 2021). This factor may help explain the presence of infective endocarditis both concomitantly with COVID-19 and after infection. The cardiological implications of infection with this virus are numerous, including endocardial involvement (Basso et al., 2020; Egas et al., 2021).

In continuation of the above, the reduced size of the reporting of post-COVID-19 endocarditis cases in pediatrics can also be attributed to the omission of objectifying the respiratory pathology from this intercausality. Although initially we attributed this mistake exclusively to the lack of testing, we identified in the literature and reports attesting the positivity of SARS-CoV-2 mRNA samples in the endomyocardial biopsies collected from patients tested negative for COVID-19 in the nasopharyngeal swab. These cases are reported on the adult population, and further research is needed to evaluate the possibility of extrapolation of the data in pediatrics (Wenzel et al., 2020). The hypothesis of missed diagnosis at the pediatric age, even more so in the adolescent population, is strengthened by the existence of numerous reports of post-COVID-19 endocarditis among patients between 19–25 years old. These cases were identified both on structurally normal heart and on affected heart (e.g., bicuspid aortic valve, coarctation of the aorta, rheumatic heart disease), among the incriminated pathogens finding *Staphylococcus warneri, Staphylococcus aureus* and *Streptococcus gordonii* (Alizadehasl et al., 2020; Toth et al., 2020; Arbune et al., 2021; Gelman et al., 2022). Other uncertainties arise from the existence of cases of infective endocarditis in the pediatric population, during the reference period (related to the peak of the pandemic), in which the performance/results of the certifying analyzes of the acute infection with the SARS-CoV-2 virus are not mentioned (Şahiner et al., 2021). We admit that there are some discrepancies between the adolescent population and the young adult, however we consider it opportune to research the differences in incidence in the reporting of endocarditis between the two.

To keep the scientific balance in balance, we briefly discuss the case of a young adult (20 years old, male), with no pre-existing conditions, who developed non-infectious endocarditis and myocarditis after vaccination with mRNA COVID-19 (Moderna). This can be an unwanted consequence of vaccination. Cardiac damage was manifested subjectively by chest pain 2 days after the second dose and low fever, the test for COVID-19 being negative. Biologically, an increase in troponin T, creatine kinase and C-reactive protein was observed. Electrocardiography showed ST-segment elevation in leads I, aVL, and V2–6. Echocardiography identified an anterior wall motion abnormality in the left ventricle. Angiography with computed tomography did not demonstrate coronary anomalies, but six minutes after contrast administration a delayed enhancement was observed in the left ventricular anterior wall, an aspect suggestive of acute myocarditis. Right ventricular endomyocardial biopsy revealed endocardial thickening consisting of myeloperoxidase-positive erythrocytes and neutrophils. In parallel, a focal infiltration of the endocardium with mononuclear cells (natural killer cells) was also observed (Aikawa et al., 2022).

Decreased immunity and altered defense mechanisms are pathways that allows bacteria, fungi or, more rarely, viruses, including SARS-CoV-2, to reach the endocardium and modify it in such a way as to allow the evolution towards infectious endocarditis. Moreover, increased endocardial inflammation leads to increased endocardial fragility. This mechanism makes the inflamed endocardium an entry point for pathogens, which through local tissue invasion leads to endocarditis. The anatomopathological results presented by Dolhnikoff M. et al. revealed the presence of RNA from the SARS-CoV-2 structure in the cardiac tissue. Thus, it can be stipulated that infection with SARS-CoV-2 can cause infective endocarditis through both immune and inflammatory mechanisms, as well as through direct damage to cardiac tissue. Although it is clear that the inflammatory and immunodeficiency phenomena produced by this infection may be the substrate of infective endocarditis, further studies are needed to unequivocally confirm this hypothesis (Dolhnikoff et al., 2020).

Based on currently available data, the COVID-19 is both a predisposing factor and a determinant factor, as it can have an indirect or direct effect on the development of infective endocarditis. As a predisposing factor, COVID-19 can weaken the immune system and make individuals more susceptible to infections, including bacterial or fungal aggression. Endothelial damage is a direct consequence of COVID-19 because SARS-CoV-2 can cause endothelial dysfunction and damage in blood vessels throughout the body. Endothelial injury can compromise the heart valves’ typical protective barriers, thereby fostering the growth of bacteria or fungi and allowing the onset of infective endocarditis. Hypercoagulable state and blood clot formation, which can harm the heart valves and increase the risk of infective endocarditis, can be considered an indirect consequence of COVID-19. Invasive medical treatments, such as oro-tracheal intubation and central venous catheter implantation, could also spread germs into COVID-19 patients’ blood stream and hasten the onset of infective endocarditis (Bele et al., 2023).

MRI can find valvular vegetation characteristics of infectious endocarditis (Bhuta et al., 2023). Additionally, the identification of delayed enhancement indicative of inflammation of the endothelial cells of the cardiovascular structures can aid in the diagnosis and planning of infectious endocarditis therapy in the absence of vegetations - data validated in both the pediatric and adult populations (Dursun et al., 2015). Compared to echocardiography, cardiac magnetic resonance (CMR) is more accurate at detecting the existence, quantity, and size of vegetations. By allowing for precise tissue identification, CMR distinguishes vegetations from other masses (Elagha et al., 2020). The identification of hidden abnormalities, such as edema, myocarditis, diffuse subendocardial fibrosis, and myocardial infarction, is also made possible by CMR. Due to its superior spatial resolution, CMR is the best method for characterizing heart tissue (Mavrogeni et al., 2017). Through a variety of mechanisms, including, ischemia, direct viral invasion, oxygen supply-demand imbalance, cytokine storm, and immunological dysregulation, SARS-CoV-2 infection can cause myocardial injury (Giustino et al., 2020). The results of cardiac MRI in individuals with COVID-19 myocarditis are consistent with those from other myocarditis sources. It contains basal to mid-inferolateral and inferior segments with linear subepicardial and mid-wall late gadolinium enhancement (Kim et al., 2021). Depending on when post-contrast imaging is performed, late gadolinium enhancement in myocarditis mostly shows inflammation with or without fibrosis (Mavrogeni et al., 2016).

When COVID-19 is identified in a patient with underlying cardiac illness, the patient’s infection is treated similarly in the general population. Currently, supportive care is the main course of action for COVID-19, with the goal of providing adequate oxygenation and nutrition, as well as reducing symptoms (Escosa-García et al., 2020; Hanff et al., 2020; Miao et al., 2020). Cardiovascular events should be managed according to the most recent guidelines for each condition (Rodriguez-Gonzalez et al., 2020). Thus, infective endocarditis and infection with SARS-CoV-2 should be treated as two different entities, but in a rational way considering the interactions between the drugs used to treat each of these diseases.

The prevention of viral endocarditis in children involves good hygiene practices such as regular hand washing, avoiding contact with people who have contagious illnesses, and maintaining good oral health to prevent local infections from spreading to the heart (Duval et al., 2019; Schriber et al., 2021). It is also important to ensure that children receive appropriate vaccinations to prevent infections that can lead to viral endocarditis (Baddour, 1999). There are currently no reports in the literature on the use of SARS-CoV-2 vaccines to prevent the infective endocarditis caused by this virus. Additional research is required to determine the link between COVID-19 and pediatric infective endocarditis, as well as the effectiveness of current vaccinations as a preventative measure.

## 4 Conclusions

Our main finding is attributed to the involvement of SARS-CoV-2 in the pathophysiology of infectious endocarditis in children with risk factors or previously healthy children. The virus appears to have catastrophic effects not only on the lungs but also on the cardiovascular system, leading to devastating illnesses for children. The incriminated mechanisms are either direct damage (in viral endocarditis) or facilitate the action of other pathogens specific to endocarditis by disrupting the immune system. The present work includes, however, simultaneously, two different cohorts of patients in terms of the pathogen causing cardiac damage. The distinctive characteristic of the cases is clinically marked by the progressive aggravation, recurrence of symptoms in children with a complex medical history, in the case of bacterial superinfection, in contrast to an acute, louder clinical picture, started in an apparent state of health, in the case of endocarditis due to unknown, presumed viral. Paraclinical, in the case of bacterial/fungal endocarditis, chronic antibodies against COVID-19 were detected. In the rest of the situations, specifying the positivity of anti-COVID 19 antibodies without classifying them as dependent on their type can be considered a reporting bias. Another bias in reporting and assessment can be considered the absence of specification of vaccinated/unvaccinated status if the patient falls within the optimal age range for this. Thus, to decrease the morbidity and mortality rate associated with pediatric endocarditis, future research directions must focus on highlighting the differences in the incidence of cardiac lesions among patients with preexisting cardiac anomalies, compared to children with a structurally intact heart. It is also necessary to consider the possible confounding factors resulting from the systemic damage caused by the confrontation with a chronic, sometimes disabling pathology in the first category. Although it is almost certain that the practical purpose of the research will no longer apply to patients infected with SARS-CoV-2, COVID-19 may represent a cornerstone in the analysis of cardiac involvement from viral infections in children. However, prudence and interest in analysis can increase future responsiveness, thereby reducing morbidity from future epidemics/pandemics.

## Author contributions

AL: Conceptualization, Investigation, Writing – original draft. AN: Validation, Visualization, Writing – review & editing. PB: Investigation, Software, Writing – original draft. EJ: Investigation, Methodology, Writing – original draft. IS: Methodology, Validation, Writing – review & editing. OF: Investigation, Software, Writing – original draft. II: Validation, Visualization, Writing – review & editing. MB: Software, Validation, Writing – review & editing. DS: Validation, Visualization, Writing – review & editing. DM: Software, Validation, Writing – review & editing. RR: Investigation, Software, Writing – original draft. RS: Validation, Writing – review & editing. CS: Validation, Writing – review & editing. VL: Writing – review & editing, Methodology, Supervision.
